# Effect of a tDCS electrode montage on implicit motor sequence learning in healthy subjects

**DOI:** 10.1186/2040-7378-3-4

**Published:** 2011-04-17

**Authors:** Eun Kyoung Kang, Nam-Jong Paik

**Affiliations:** 1 Department of Rehabilitation Medicine, Seoul National University Bundang Hospital, Seongnam, South Korea; 2 Department of Rehabilitation Medicine, Seoul National University College of Medicine, Seoul, South Korea

**Keywords:** TDCS, Motor learning, Cortical stimulation, Implicit learning

## Abstract

**Background:**

This study was undertaken to test the hypothesis that a combination of excitatory anodal transcranial direct current stimulation (tDCS) to the contralateral motor cortex and inhibitory cathodal tDCS to the ipsilateral motor cortex of the motor performing hand (Bi-tDCS) would elicit more implicit motor sequence learning than anodal tDCS applied to the contralateral motor cortex alone (Uni-tDCS).

**Methods:**

Eleven healthy right-handed adults underwent a randomized crossover experiment of Uni-tDCS, Bi-tDCS, or sham stimulation. Subjects performed a 12-digit finger sequence serial reaction time task with the right hand at baseline (Pre), at immediately (Post 1), and 24 hours after stimulation (Post 2). The ratios of reaction times of predetermined repeating sequence versus random sequence were subjected to statistical analysis.

**Results:**

The paired *t *test showed that reaction time ratios were significant decreased by all stimulation types at Post 1 versus Pre (P < 0.01). However, mean reaction time ratios showed a significant decrease after Uni-tDCS (P < 0.01) and Bi-tDCS (P < 0.01), but only a marginal decreased after Sham (P = 0.05) at Post 2, which suggests that motor sequence learning is consolidated by Uni-tDCS and Bi-tDCS, but only partially consolidated by sham stimulation. No significant differences were observed between Uni-tDCS and Bi-tDCS in terms of in reaction time ratios at Post 1 or 2.

**Conclusions:**

No significant difference was found between Uni-tDCS and Bi-tDCS in terms of induced implicit motor sequence learning, but tDCS led to greater consolidation of the learned motor sequence than sham stimulation. These findings need to be tested in the context of stroke hand motor rehabilitation.

## Background

Recently transcranial direct current stimulation (tDCS), a non-invasive brain stimulation technique has been applied to facilitate skill acquisition and motor learning [1-4].

TDCS modulates cortical excitability in a polarity dependent manner, that is, anodal tDCS increases but cathodal tDCS decreases cortical excitability at stimulated sites [5,6]. Furthermore, anodal tDCS applied to the contralateral motor cortex of the motor performing hand [1,3] or cathodal tDCS applied to the ipsilateral motor cortex [7,8] have been reported to improve motor performance in healthy subjects. This concept has also demonstrated in stroke patients. In these studies, anodal tDCS applied to the affected motor cortex [9,10] or cathodal tDCS applied to the unaffected motor cortex [10,11] to diminish inter-hemispheric trans-callosal inhibition [7,12] was shown to improve affected hand motor performance.

Given the findings of the above-mentioned reports, it is possible that a combination of anodal tDCS to the contralateral motor cortex and cathodal tDCS to the ipsilateral motor cortex of the motor performing hand would improve motor performance more than the application of anodal tDCS to the contralateral motor cortex alone.

Therefore, the purpose of this study was to test the above hypothesis using an implicit finger-sequence learning paradigm [13] in healthy subjects. Furthermore, functional recovery after stroke is a motor relearning process [14,15], and thus, it was hoped that the results of this study may be applicable to stroke patients.

## Methods

### Subjects

Eleven healthy young adults (three males, age 26.3 years ± 3.6 S.D.) without any medical or neurological disease participated in this study. All were right handed, as determined by the Edinburgh Handedness Inventory [16]. The experimental protocol was approved by the Institutional Review Board at our hospital and written informed consent was obtained from all subjects.

### Experimental design

**Figure 1:**
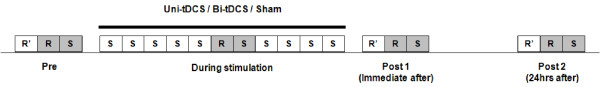
**Experimental design**. Motor sequence performance improvement was measured by calculating the ratio of reaction times for the predetermined repeating sequences and a random sequence (S/R block) at shaded blocks. R' = familiarizing random sequence block; R = random sequence block; S = predetermined repeating sequence block.

After being familiarized with the experimental setting, each of the 11 subjects underwent a randomized crossover experiment of Uni-tDCS, Bi-tDCS, or sham stimulation separated by at least 48 hours. Orders of stimulation conditions were counterbalanced (Figure 1).

TDCS was delivered through two saline-soaked, sponge electrodes (25 cm^2^) using a constant-current stimulator (Phoresor^®^ΙΙ PM850; IOMED^® ^Inc., Salt Lake City, Utah) as previously described [9]. Although Phoresor^®^ΙΙ PM850; IOMED^® ^Inc. is not designed for tDCS, it has been widely used for tDCS studies. This device can control current intensity, duration, and ramp up time [17].

First, we placed three electrodes over C3 (corresponding to the left M1), C4 (corresponding to the right M1) of the international 10-20 EEG system, and the right supra-orbital region. For Uni-tDCS (2 mA for 20 minutes) and sham stimulation (2 mA for 1 minute), we used an anode electrode over C3 and a cathode over the right supraorbital region, and for Bi-tDCS (2 mA for 20 minutes) we used an anode over C3 and a cathode over C4.

The current was slowly increased to 2 mA from the onset of stimulation in a ramp-up like fashion over 30 sec. For real stimulation, the switch was toggled up and down for an additional 30 sec to match the sham procedure, and the current was then maintained at 2 mA for the remainder of the 20 min, whereas during sham stimulation sessions the current was slowly tapered down to zero over 30 sec. This procedure has been demonstrated to prevent subjects differentiating between real and sham stimulation [9,18]. We selected C3 and C4 of the international 10-20 EEG system for stimulation because it has been reported that the primary motor cortex (M1) mediates implicit motor sequence learning [19], and because a neuroimaging study showed that C3 and C4 correspond to the left and right M1 [20]. However, in the present study, stimulation may have extended beyond M1 due to the large electrode size used.

The tDCS procedures were administered by a separate investigator who did not participate in outcome measurements or data analysis. Therefore, the subjects and the investigator who determined outcome measures were unaware of the intervention type.

### Serial reaction time task

We used a serial reaction time task (SRTT) as an outcome measure. The SRTT is a simple task that provides a measure of implicit motor skill learning [21]. Subjects performed a total of 20 blocks of key presses with their right hands, and each block was composed of 10 repetitions of a 12-digit length sequence (Figure 1). Subjects were seated in front of a computer screen and asked to press the key corresponding to the location of asterisks with 4 fingers (2^nd ^- 5^th^) of the right hand as quickly and as accurately as possible. The task was designed using Superlab pro v.4.0 software (Cedrus Corporation, San Pedro, CA).

After familiarization using a random block (R' in Figure 1), subjects were presented with random (R in Figure 1) or predetermined repeating sequence blocks (S in Figure 1) separated by resting 30 sec periods. Next blocks were presented when all keys presses were correct.

For the R' and R blocks, an asterisk appeared randomly in one of four locations on a computer screen, whereas an asterisk appeared in a predetermined repeating sequence in an S block. We used three predetermined repeating sequence S blocks (3-4-2-1-2-4-1-3-4-2-1-3/2-4-1-3-2-1-2-1-3-4-3-4/1-2-1-4-2-3-2-4-3-1-4-3), one for each of the three stimulation types (Uni-tDCS, Bi-tDCS, or sham stimulation) in a randomly selected manner. These R'-R-S blocks were presented at baseline (Pre), immediately (Post 1), and 24 hours after stimulation (Post 2). During stimulation, subjects practiced using the same predetermined repeating sequence S blocks (block 4-8 and 10-14) interrupted by one R block. The ratios of reaction times for the predetermined repeating sequence and the random sequence (shaded S block/R block in Figure 1) were used as an outcome measure of motor sequence performance improvements by practice.

Prior to each session, subjects described their levels of attention, perceived general fatigue, hand fatigue, and task difficulty using a numeric rating scale (range 0 ~ 10; 0 = lowest, 10 = highest).

### Data analysis

The mean response time per each trial was calculated to quantify motor sequence performance improvements (motor sequence learning) achieved by repeated practice. The ratios of reaction times of predetermined repeating sequence per random sequence (shaded S block/R block in Figure 1) at Pre, Post 1, and Post 2 relative to baseline were analyzed using the paired *t *test for each stimulation type (Uni-tDCS versus Bi-tDCS versus sham stimulation) to demonstrate the motor sequence learning effect.

## Results

ANOVA_RM _revealed no effect of INTERVENTION_Uni-tDCS, Bi-tDCS, Sham_, TIME_Pre.Post1, Post 2 _or INTERVENTION_Uni-tDCS, Bi-tDCS, Sham_×TIME_Pre.Post1, Post 2 _interaction on subjects' perceived attention and general fatigue (P > 0.05). But, there was a significant effect of TIME_Pre.Post1, Post2 _on hand fatigue and task difficulty, which suggested that subject perceived hand fatigue increased and task difficulty decreased immediate after practice blocks (Table 1).

**Table 1:** Subject perceived levels of attention, general fatigue, hand fatigue, and task difficulty (rated using numeric 0~10 rating scales; 0 = lowest, 10 = highest).

	Stimulation type									ANOVA_RM_ P-value		
	
	Uni-tDCS			Bi-tDCS			Sham			Interv	Time	Interv X Time
	Pre	Post 1	Post 2	Pre	Post 1	Post 2	Pre	Post 1	Post 2			
Attention	5.2 ± 1.3	5.0 ± 2.3	5.8 ± 1.6	5.6 ± 1.3	5.2 ± 2.1	4.8 ± 2.3	5.8 ± 2.0	5.7 ± 2.0	5.2 ± 1.4	0.598	0.764	0.431
Fatigue	4.9 ± 1.1	4.3 ± 1.8	5.6 ± 2.0	4.6 ± 1.6	4.2 ± 1.5	4.0 ± 1.9	4.7 ± 2.2	5.1 ± 1.4	4.5 ± 2.4	0.168	0.464	0.250
Hand fatigue	5.9 ± 2.3	5.1 ± 2.2	5.7 ± 1.7	6.0 ± 2.0	5.3 ± 2.2	5.1 ± 2.1	5.7 ± 2.3	5.5 ± 2.5	5.7 ± 2.4	0.822	0.046	0.619
Task difficulty	5.7 ± 1.8	5.4 ± 2.3	6.2 ± 1.8	6.0 ± 2.0	4.7 ± 1.7	5.2 ± 2.0	6.0 ± 2.0	5.7 ± 2.5	5.9 ± 2.2	0.295	0.008	0.242

**Figure 2:**
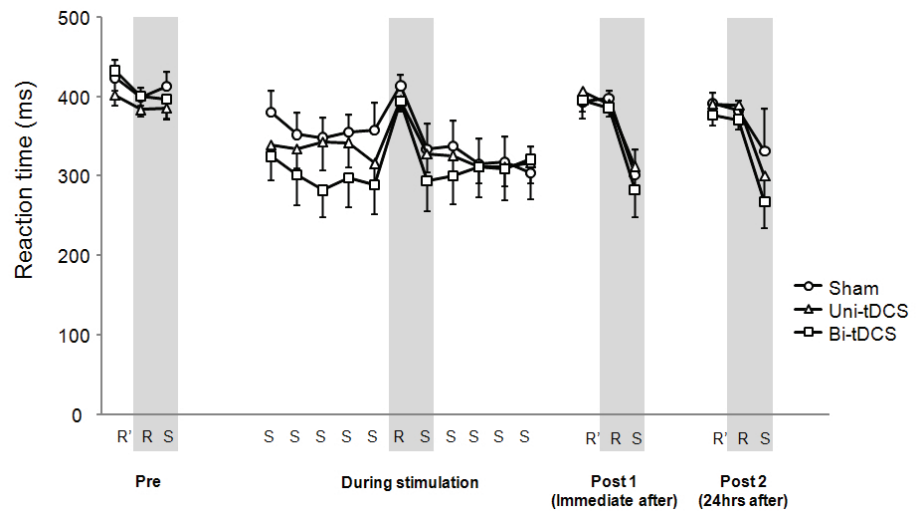
**Serial reaction times for each stimulation type**. Note that mean reaction time was shortened during the predetermined repeating sequence blocks but returned to baseline level during the random sequence blocks regardless of stimulation type.

For each stimulation type, mean reaction time shortened during the predetermined repeating sequence blocks, but return to the baseline level during the random sequence blocks (Figure 2).

The mean S/R ratio (ratio of reaction time for a predetermined repeating sequence versus a random sequence) at Pre did not differ significantly for the three stimulation types (P = 0.57 by one way ANOVA).

When comparing sham and Uni-tDCS or sham and Bi-tDCS at Post 2 using the paired t test, no significant differences were found (Sham vs. Uni-tDCS, P = 0.49; Sham vs. Bi-tDCS, P = 0.19). Furthermore, no significant S/R ratio difference was observed between Uni-tDCS and Bi-tDCS at Post 2. ANOVA also revealed no significant differences between stimulation types at Post 2 (P = 0.65).

**Figure 3:**
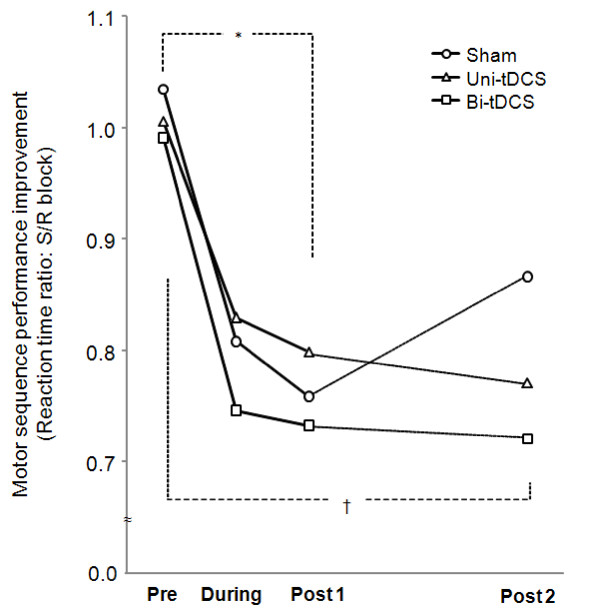
**Motor sequence performance improvement**. The Y axis represents the ratios of the reaction times of the predetermined repeating sequence versus a random sequence (shaded S versus R blocks in Figure 1). The asterisk (*) represents P < 0.05 between Pre and Post 1 sessions for all stimulation types by the paired t test, which suggests motor sequence learning occurred at immediately after stimulation regardless of stimulation type. Cross(+) represents P < 0.05 between Pre and Post 2 sessions for Uni-tDCS and Bi-tDCS, but not for Sham stimulation by the paired t test, which suggests that motor sequence performance improvements were maintained after Uni-tDCS and Bi-tDCS, but not after Sham stimulation.

We believe these negative findings were caused by small subject numbers. Therefore, we performed paired t-testing between Pre and Post1 or Pre and Post 2 for each stimulation type. We found that S/R ratio significantly decreased for all stimulation types at Post 1 (P < 0.01), but at Post 2, this reduction was significant after Uni-tDCS (P < 0.01) and Bi-tDCS (P < 0.01), and only marginally significant after Sham (P = 0.05), which suggested that motor sequence performance improvement was maintained by Uni-tDCS and Bi-tDCS, but only partially by sham stimulation. However, no significant S/R ratio differences were observed between Uni-tDCS and Bi-tDCS at Post 1 and Post 2 (Figure 3).

## Discussion

In this study, we evaluated the combining effect of anodal tDCS applied to the contralateral motor cortex and cathodal tDCS applied to the ipsilateral motor cortex (Bi-tDCS) on the implicit motor learning process, and compared this with the effect of anodal tDCS applied to the contralateral motor cortex alone (Uni-tDCS). We found that combined bilateral stimulation did not improve implicit motor learning more than unilateral stimulation, but that Bi-tDCS and Uni-tDCS did improve implicit motor learning more than sham stimulation.

Initially we hypothesized that decreasing inter-hemispheric trans-callosal inhibition from non-dominant to dominant M1 by right hemisphere cathodal tDCS in combination with increasing the excitability of dominant M1 by left hemisphere anodal tDCS would improve implicit motor learning more than increasing the excitability of dominant M1 by left hemisphere anodal tDCS alone. However, our findings did not support this hypothesis, although it should be noted that Bi-tDCS showed a tendency to more improve implicit motor learning than Uni-tDCS, as is shown by the raw data presented in Figures 1 and 2.

Recently, Vines et al. [22] found that Bi-tDCS improved motor performance of the non-dominant hand more than Uni-tDCS in healthy subjects, whereas in the present study only a weak trend was found. We believe that this discrepancy may have been caused by the different task paradigm used or the use of the non-dominant hand, because in this previous study a 5 digit sequence and non-dominant left hands were used. It is possible that dominant hands might have already reached a ceiling prior to stimulation [1,7,8], or that interhemispheric inhibition from non-dominant to dominant hemisphere might be trivial as compared with inhibition from dominant to non-dominant hemisphere [22-24]. It is also probable that healthy young subjects are more likely to display the ceiling effect than older subjects or stroke patients in implicit motor learning process. It is also possible that our study was underpowered due to small number of subjects recruited.

Although the clinical applications of tDCS have expanded, the effects of electrode montages have not been well established. One unique aspect of tDCS application is the use of an electrodes pair. The belief that tDCS increases excitability just at the stimulating site under the anode and decreases excitability under the cathode is changing. Now it is generally believed that tDCS has both a regional effect on the cortex underlying electrodes and a remote effect on brain regions between electrodes [25-27]. Moreover, recently Moliadze et al. addressed the role of the "return" electrode position on tDCS induced excitability changes under an the "active" electrode using a computer model, and showed that the position and size of the ''return" electrode affects the electric field distribution across the entire cortex, and the electric field distribution in cortex directly under the "active"electrode [28].

According to this view, the anodal effects on C3 during Uni-tDCS and Bi-tDCS differed in the present study, because the return cathode was positioned over the contralateral supraorbital region for Uni-tDCS and over C4 for Bi-tDCS. We used this Bi-tDCS electrode montage hoping to simultaneously up-regulate excitability of the motor cortex over C3 (anodal stimulation), and to down-regulate excitability of the motor cortex over C4 (cathodal stimulation). Recently Lindenberg et al. [29] also used the same Bi-tDCS electrode montage used in the present study.

Extracephalic electrode montages offer another approach [30]. According to this method one electrode is placed over the cortex and the other over an extracephalic region, such as, a shoulder or mastoid process. It would be interesting to compare Uni-tDCS and Bi-tDCS using this extracephalic electrode montage in the future. However, in the present study, we could not exclude the possibility that during Uni-tDCS, the reference cathode on the right supraorbital region, which corresponds to the right prefrontal cortex, might have had some beneficial effect on implicit motor sequence learning, which would have diluted the additive effect of Bi-tDCS over Uni-tDCS. Additional experimental studies are required to investigate the effects of various electrode montages on the effects of tDCS.

Another possibility is that the electrode size over M1 was large enough to cover the pre-motor cortex, which also would have had a diluting effect on Bi-tDCS versus Uni-tDCS. In a neuroimaging study, it was found that finger sequence performance recruits the pre-motor and supplementary motor cortex as well as the primary motor cortex [31], although we only intended to stimulate M1 as performed in a previous study [3], in which it was shown that finger sequence performance task results can be influenced by modulating M1 activity.

In the present study, the reaction times of predetermined repeating sequence in the SRTT decreased regardless of stimulation type, whereas the reaction times of random sequences did not, which implies that implicit motor learning had occurred during training. However decreases in reaction times immediately after sham stimulation tended to diminish at 24 hours, but were maintained after Uni-tDCS and Bi-tDCS, which suggest that tDCS might consolidate implicit motor learning more than Sham stimulation, which is in accordance with a previous report [4]. Our results also reveal that tDCS mainly affected motor performance speed rather than accuracy.

Subject attention levels could also have contributed to SRTT results, though these were similar across sessions as determined by our numerical rating scale, and thus, we believe that subject attention level differences were adequately taken into account.

TDCS is easily administered, comfortable for patients, relatively inexpensive and can be administered in combination with rehabilitative training [18], and for these reasons was recently introduced as an adjuvant strategy for hand motor rehabilitation after stroke [9,10]. Our results might be relevant to stroke hand motor rehabilitation, although its relevance is limited by potential differences in the implicit motor learning process between healthy subjects and stroke patients.

## Conclusions

In conclusion, no significant difference was observed between Uni-tDCS and Bi-tDCS in terms of inducing implicit motor sequence learning, although both Uni-tDCS and Bi-tDCS led to greater consolidation of the learned motor sequences than sham stimulation. The findings of the present study need to be tested in the context of stroke hand motor rehabilitation.

## Competing interests

The authors declare that they have no competing interests.

## Authors' contributions

NJP designed the study and EKK carried out the study and analyzed the data. Both authors drafted the manuscript, and finally read and approved the last version manuscript.
